# PTH1R Mutants Found in Patients with Primary Failure of Tooth Eruption Disrupt G-Protein Signaling

**DOI:** 10.1371/journal.pone.0167033

**Published:** 2016-11-29

**Authors:** Hariharan Subramanian, Frank Döring, Sina Kollert, Natalia Rukoyatkina, Julia Sturm, Stepan Gambaryan, Angelika Stellzig-Eisenhauer, Philipp Meyer-Marcotty, Martin Eigenthaler, Erhard Wischmeyer

**Affiliations:** 1 Institute of Clinical Biochemistry and Pathobiochemistry, University of Wuerzburg, Wuerzburg, Germany; 2 Institute of Experimental Cardiovascular Research, University Medical Centre Hamburg-Eppendorf, Hamburg, Germany; 3 Institute of Physiology, AG Molecular Electrophysiology, University of Wuerzburg, Wuerzburg, Germany; 4 Sechenov Institute of Evolutionary Physiology and Biochemistry, Russian Academy of Sciences, St. Petersburg, Russia; 5 Department of Cytology and Histology, St. Petersburg State University, St. Petersburg Russia; 6 Department of Orthodontics, University Clinic of Wuerzburg, Wuerzburg, Germany; 7 Department of Orthodontics, University Clinic of Goettingen, Goettingen, Germany; University of Pittsburgh School of Medicine, UNITED STATES

## Abstract

**Aim:**

Primary failure of tooth eruption (PFE) is causally linked to heterozygous mutations of the parathyroid hormone receptor (PTH1R) gene. The mutants described so far lead to exchange of amino acids or truncation of the protein that may result in structural changes of the expressed PTH1R. However, functional effects of these mutations have not been investigated yet.

**Materials and Methods:**

In HEK293 cells, PTH1R wild type was co-transfected with selected PTH1R mutants identified in patients with PFE. The effects on activation of PTH-regulated intracellular signaling pathways were analyzed by ELISA and Western immunoblotting. Differential effects of wild type and mutated PTH1R on TRESK ion channel regulation were analyzed by electrophysiological recordings in *Xenopus laevis* oocytes.

**Results:**

In HEK293 cells, activation of PTH1R wild type increases cAMP and in response activates cAMP-stimulated protein kinase as detected by phosphorylation of the vasodilator stimulated phosphoprotein (VASP). In contrast, the PTH1R mutants are functionally inactive and mutant PTH1R/Gly452Glu has a dominant negative effect on the signaling of PTH1R wild type. Confocal imaging revealed that wild type PTH1R is expressed on the cell surface, whereas PTH1R/Gly452Glu mutant is mostly retained inside the cell. Furthermore, in contrast to wild type PTH1R which substantially augmented K^+^ currents of TRESK channels, coupling of mutated PTH1R to TRESK channels was completely abolished.

**Conclusions:**

PTH1R mutations affect intracellular PTH-regulated signaling *in vitro*. In patients with primary failure of tooth eruption defective signaling of PTH1R mutations is suggested to occur in dento-alveolar cells and thus may lead to impaired tooth movement.

## Introduction

Parathyroid hormone (PTH), PTH-related peptide (PTHrP) and parathyroid hormone receptor type 1 (PTH1R) play a key role in regulation of bone remodeling and plasma calcium levels as reviewed by Taylor et al. [1]. Various autosomal dominant or recessive mutations in the PTH1R gene have been identified to be associated with different diseases. The most severe clinical disease is a complete loss of PTH1R function resulting in lethal Blomstrand chondrodysplasia characterized by advanced endochondral bone maturation and premature ossification of all skeletal elements [2].

In contrast to this homozygous disease, we and others have described the incidence of heterozygous PTH1R mutations which may inactivate PTH1R function in Primary Failure of Tooth Eruption (PFE) (MIM #125350). PFE is a rare autosomal, non-syndromic disorder with incomplete eruption of mainly posterior teeth and growth deficiency of the alveolar process in the affected region [3, 4]. The developing teeth remain below the occlusion level resulting in a severe lateral open bite situation. Furthermore, movement of the teeth by orthodontic treatment results in fusion of the dental cement with the surrounding bone making further tooth movement impossible [5].

The genetic defects underlying PFE were initially identified by genetic screening in affected families [3]. While the first reported 3 mutations (c.463G>T, c.543+1G>A, c.1050-3C>G) in patients with PFE were predicted to generate loss-of-function proteins [3], the spectrum of PTH1R mutations by now has been expanded by several investigators showing the occurrence of more than 40 potentially pathogenic mutations [4, 6–8]. Furthermore, occurrence of sporadic cases of PTH1R mutations causing PFE have been identified by exome resequencing [9]. The PTH1R mutations identified so far are heterogenous and may result in proteolytic degradation of the PTH1R precursor protein, truncation of the PTH1R protein or single amino acid exchanges within the complex architecture of the receptor. However, until now no functional data on the molecular and cellular effects of the PFE related mutations in eukaryotic cells exist.

The PTH1R gene encodes a secretin-like class II G protein-coupled receptor precursor that is processed into a mature receptor protein of a 593 amino acids. The PTH1R receptor belongs to the group of seven-helical-transmembrane receptors and mainly consists of a large extracellular loop with a PTH binding site, seven transmembrane, helically shaped segments and an intracellular signaling domain. PTH1R is highly expressed in osteoblasts and renal tubular cells and regulates calcium homeostasis and bone formation. In addition to PTH agonist, PTH1R is activated by PTHrP [10,11], a paracrine hormone that also regulates bone development and epithelial-mesenchymal interactions in developing teeth.

Signal transduction of PTH1R is mediated by the intracellular domain which couples to G_αs_-proteins and thus increases the activity of adenylate cyclase (AC). This signaling pathway is well characterized in osteoblasts [12], leading to increase of intracellular cAMP and accordingly to cAMP-dependent activation of enzymes such as protein kinase A (PKA). PKA subsequently phosphorylates various signaling proteins. One major target protein is the vasodilator-stimulated phosphoprotein (VASP) regulating cell adhesion and motility [13]. Alternatively, the PTH1R is able to activate the G_q11_/phospholipase C/ calcium/ PKC pathway [14]. One target of this pathway is the tandem-pore (K_2_P) potassium channel TRESK [15,16] which is activated in a calcium-dependent manner.

In our present study, we used a cell model that allows transfection of PTH1R and its mutants to study their effect on intracellular, PTH-activated signaling pathways. For initial experiments, we selected two known mutations from patients with PFE, the PTH1R/Trp339stop mutation resulting in a truncated protein and the PTH1R/Gly452Glu mutation with single amino acid exchange and may affect the structure of the receptor [3, 10]. Both mutants were functionally inactive and also in heterozygous circumstances strongly interfered with the activation of physiological PTH1R receptor signaling pathways.

## Materials and Methods

### Ethics statement

The study was approved by the Ethic Committee of the University Clinic of Wuerzburg, vote 103/04 and 192/11.

### Materials

Forskolin was purchased from Sigma Aldrich (Munich, Germany). PTH was obtained from Tocris (Wiesbaden-Nordenstadt, Germany). Antibodies against Phospho-VASP^Ser157^, Actin, and GAPDH were from Cell Signaling (Frankfurt/Main, Germany) and were used in a 1:1000 dilution. Antibody against PTH1R was from Thermo Scientific (Bonn, Germany) and was used in a 1:1000 dilution.

### PTH1R constructs

Full-length human PTH1R cDNA was a gift from Dr. Lohse, Institute of Pharmacology of the University of Wuerzburg. The mutants (c.G1016A (p.Trp339stop) and c.G1355A (p.Gly452Glu) were generated by QuikChange site-directed mutagenesis Kit (Stratagene, La Jolla, USA) following the manufacturer’s protocol. To detect PTH1R in membrane fractions of oocytes myc-tag was fused to the C-terminus of all constructs, respecting the premature stop codon of the PTH1R/Trp339stop mutant. For biochemical assays in HEK293 cells cDNAs were subcloned into pcDNA3 vector. The oocyte expression vector pSGEM was used to express receptors and K^+^ channels for electrophysiological recordings and Western blots in *Xenopus laevis* oocytes. Cloning of mouse K_2_P channel TRESK has been previously described [15,16]. For confocal imaging PTH1R wild type and PTH1R/Gly452Glu mutant were subcloned into pEYFP-N1 vector to express PTH1R with c-terminal YFP.

### Extraction and immunoblotting of oocyte membranes

*Xenopus laevis* oocytes injected with *in vitro* transcripts of myc-tagged PTH1R wild type, PTH1R/Gly452Glu PTH1R/Trp339stop or H_2_O were incubated for 48 h in ND 96 solution (96 mM NaCl, 2 mM KCl, 1 mM MgCl_2_, 1 mM CaCl_2_, 5 mM HEPES, pH 7.4) and subsequently homogenized by repeated pipetting in solubilization buffer (10mM HEPES, pH 7.9, 1mM MgCl_2_, 83mM NaCl, 0,5mM PMSF, protease inhibitor cocktail complete [Roche]). Plasma membranes from homogenates of 25 oocytes were isolated by sequential centrifugations at 2x 1000g (10 min) and 10000g (20 min) at 4°C. Precipitates of the final centrifugation step were solubilized in modified Laemmli loading buffer (126 mM Tris/HCl pH 6,8, 300 mM DTT, 6% SDS, 10% Glycerol 0,2% bromophenol blue). Equal amounts of precipitated membrane fractions were subjected to PAGE and analyzed by Western immunoblots probed with monoclonal mouse anti myc-tag antibody (1:500; clone 9B11; CST, Danvers, MA). For detection HRP-conjugated goat anti mouse immunoglobulins (1:10,000; Jackson ImmunoResearch Laboratories, West Grove, PA) were applied and after washing developed with self-prepared chemiluminescence reagent (0.1 M Tris pH 8.6, 1.25 mM Luminol, 0.6 mM p-Cumaric acid, 0.01% H_2_O_2_).

### Transfection and experiments on HEK293 cells

HEK293 cells were cultured in Dulbecco’s Modified Eagle’s Medium (DMEM), supplemented with 10% fetal bovine serum. Cells were transiently transfected with PTH1R wild type and/or mutant plasmids using METAFECTENE PRO (Biontex, Martinsried/Planegg, Germany) following the manufacturer’s protocol. 24 h after transfection cells were washed with PBS and left in serum-free media for 2 h followed by stimulation with PTH or forskolin for 10 min. After stimulation, cells were lysed for Western blot analysis.

### Western blot analysis

For Western blot analysis cells were washed with PBS and lysed in 2x SDS gel loading buffer (500 μl / confluent well of 6 well plate). Cell lysates were separated by SDS-PAGE, transferred to nitrocellulose membranes followed by incubation with appropriate primary antibodies overnight at 4°C. For visualization of the signal, goat anti-rabbit or anti-mouse IgG conjugated with horseradish peroxidase were used as secondary antibodies (dilution 1:10000 each), followed by ECL detection. Blots were scanned using SilverFast software and analyzed densitometrically by NIH Image J software for uncalibrated optical density.

### cAMP measurement

cAMP level in HEK293 cells was evaluated using a cAMP EIA Kit (Cayman Chemical, Hamburg, Germany) following the manufacturer's instructions.

### Confocal imaging

PTH1R wild type and PTH1R/Gly452Glu mutant fused with YFP were expressed in HEK293 cells. 24 hours post-transfection, cells were transferred to imaging medium (144 mM NaCl, 5 mM KCl, 1 mM CaCl_2_, 1 mM MgCl_2_, 10 mM HEPES, pH = 7.3) and incubated with CellMask^™^ Deep red plasma membrane stain (Life Technologies GmbH Darmstadt, Germany) according to manufacturer’s instruction. Then cells were mounted onto 63X objective in Zeiss LSM800 microscope and the images were captured with Zeiss Axiocam.

### Electrophysiology

For heterologous gene expression in *Xenopus laevis* oocytes, capped run-off poly(A^+^) cRNA transcripts from linearized cDNA of wild type and mutated PTH-receptors and channels were synthesized and injected into defolliculated oocytes. Cells were incubated at 19°C in ND96 solution (96 mM NaCl, 2 mM KCl, 1 mM MgCl_2_, 1 mM CaCl_2_, 5 mM HEPES, pH 7.4) supplemented with 100 μg/ml gentamicin and 2.5 mM sodium pyruvate. 48–72 hours after injection two electrode voltage-clamp measurements were performed with a TURBO TEC-10 C amplifier (npi, Tamm, Germany). Stimulation and data acquisition were controlled by Pulse software (HEKA, Germany). Oocytes were placed in a small volume perfusion chamber with a constant flow of ND96 with 0.1% BSA or ND 96 with 0.1% BSA supplemented with different concentrations of PTH.

### Data analysis

All experiments were performed at least in triplicate and data shown are means ± S.D. Differences between groups were analyzed by one-way ANOVA or two-way ANOVA, respectively. p<0.05 was considered statistically significant, p<0.01 as statistically highly significant. Original data of ANOVA analysis are shown in supporting information files S1 and S2 Tables.

## Results

### PTH increases intracellular cAMP and induces cAMP/PKA-triggered VASP phosphorylation in HEK293 cells transfected with PTH1R

**Fig 1:**
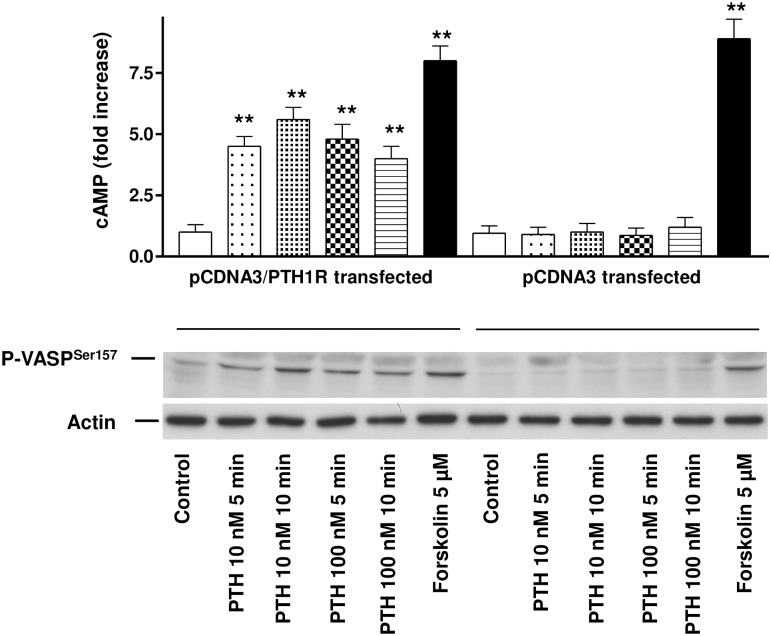
Activation of PTH1R by PTH in PTH1R transfected HEK293 cells increases intracellular cAMP and induces PKA-triggered VASP phosphorylation. The upper panel depicts cAMP increase in HEK293 cells transfected with PTH1R (pcDNA3/PTH1R transfected) or with the vector only (pcDNA3 transfected). Cells were stimulated for up to 10 min with 10 or 100 nM PTH as indicated. For PTH-independent positive control cells were stimulated by 5 μM forskolin (as depicted). Data of thee independent experiments are presented as mean ± SEM, n = 3, p< 0.05 compared to the pcDNA3 control. Phosphorylation of VASP and loading control were documented by Western immunoblots with specific antibodies as indicated in the lower panel.

In order to elucidate the efficacy of our expression system we transiently transfected HEK 293 cells with pcDNA3 or pcDNA3/PTH1R vector as described in methods. Controls and stimulation with 10 nM or 100 nM PTH was performed for up to 10 min as indicated in Fig 1. After stimulation with 10 nM PTH, HEK 293 cells transfected with the PTH1R/pcDNA3 vector responded with a 4.7 ± 0.4 and 5.8 ± 0.6 -fold cAMP increase at 5 and 10 min, respectively (Fig 1, upper panel). Increase of the PTH concentration to 100 nM did not result in a further increase in cAMP levels as shown by a 4.9 ± 0.8 and 4.3 ± 0.6 fold cAMP increase at 5 and 10 min, respectively (p<0.01, number of independent experiments (n), n = 3). Cells transfected with the empty pcDNA3 vector and stimulated by 10 nM or 100 nM PTH showed no increase in cAMP compared to unstimulated control. To demonstrate intactness of intracellular signaling pathways in the transfected cells, stimulation with 5μM Forskolin, a strong direct activator for cAMP-regulated signaling pathways was performed. Forskolin induced a 7.7 ± 0.6 or 8.3 ± 0.9 fold increase in cAMP in cells transfected with PTH1R or empty vector, respectively (p<0.01, n = 3). Increase in cAMP resulted in corresponding phosphorylation of the intracellular signaling protein VASP at the PKA-preferred phosphorylation site Serine-157 (Fig 1, lower panel). The degree of VASP phosphorylation strongly correlated with the increase of cAMP. Again, pcDNA3 transfected cells showed no increase in VASP phosphorylation above baseline, whereas the PTH-independent cAMP-elevating substance forskolin induced maximal VASP phosphorylation in both PTH1R and control transfected cells.

### PTH1R/Trp339stop and PTH1R/Gly452Glu mutants are functionally inactive and do not activate intracellular cAMP-regulated pathways

**Fig 2:**
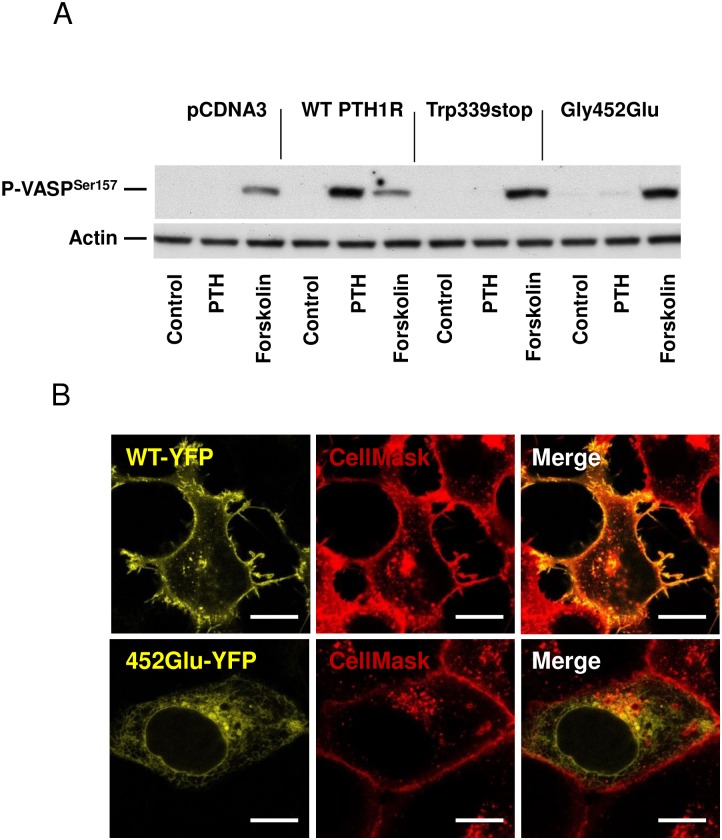
Stimulation and localization of PTH1R wild type or PTH1R mutants. (A) Extracts of stimulated (as indicated) HEK293 cells transfected with pcDNA3, pcDNA3:PTH1R wild type (WT), pcDNA3:PTH1R/Trp339stop or pcDNA3:PTH1R/Gly452Glu mutant were analyzed for VASP phosphorylation at the cAMP/PKA-preferred site Serine 157 (upper panel). Blotting against actin was used as loading control and used for quantification of VASP phosphorylation (lower panel). Representative blots of three independent experiments are shown. (B) Representative images from confocal imaging of YFP-tagged wild type (WT-YFP) and PTH1R/Gly452Glu (452Glu-YFP) in transfected HEK293 cells. CellMask deep red membrane dye was used for membrane staining. Scale bar 10 μM.

In a second set of experiments we tested the effect of two different mutations of the PTH1R protein. HEK293 cells were transfected with empty vector pcDNA3, pcDNA3:PTH1R wild type, pcDNA3:PTH1R/Trp339stop mutant or pcDNA3:PTH1R/Gly452Glu mutant plasmids as described in methods. 24 h after transfection cells were stimulated with 10 nM PTH or 10 μM forskolin (as a PTH-independent positive control of VASP phosphorylation). In cells transfected with the empty pcDNA3 vector, stimulation with PTH did not induce VASP phosphorylation, whereas stimulation with forskolin demonstrated the intactness of the intracellular cAMP signaling pathway. Transfection of HEK293 with pcDNA3:PTH1R wild type construct and stimulation with PTH increased intracellular cAMP and induced subsequent phosphorylation of PKA-induced VASP phosphorylation at Serine 157 as shown in Fig 2A (data for cAMP in supporting information file S3 Table).

In contrast, cells transfected with pcDNA3:PTH1R/Trp339stop, which codes for a truncated PTH1R protein showed no increase in cAMP and no phosphorylation of VASP after stimulation with 10 nM PTH. The second mutant PTH1R/Gly452Glu, is supposed to alter the structure of the PTH1R receptor by the exchange of glycine against glutamic acid at position 452. Again, cells transfected with the PTH1R/Gly452Glu mutant showed no increase in intracellular cAMP after PTH stimulation and no subsequent VASP phosphorylation (Fig 2A, data for cAMP in supporting information file S3 Table). Furthermore, increase of PTH concentration to 100 nM did not induce cAMP increase or VASP phosphorylation in PTH1R mutant transfected cells (data not shown). Incubation of the cells with forskolin, a PTH1R-independent elevator of cAMP, as a positive control resulted in strong cAMP/PKA-induced phosphorylation of VASP in both wild type as well as mutant transfected cells (Fig 2A). As revealed by confocal imaging, we identified co-localization of wild type PTH1R with the membrane dye deep red (CellMask), whereas the PTH1R/Gly452Glu mutant was found to be intracellular and did not colocalize with the membrane-staining dye (Fig 2B).

### Dominant negative effect of PTH1R/Gly452Glu mutant

**Fig 3:**
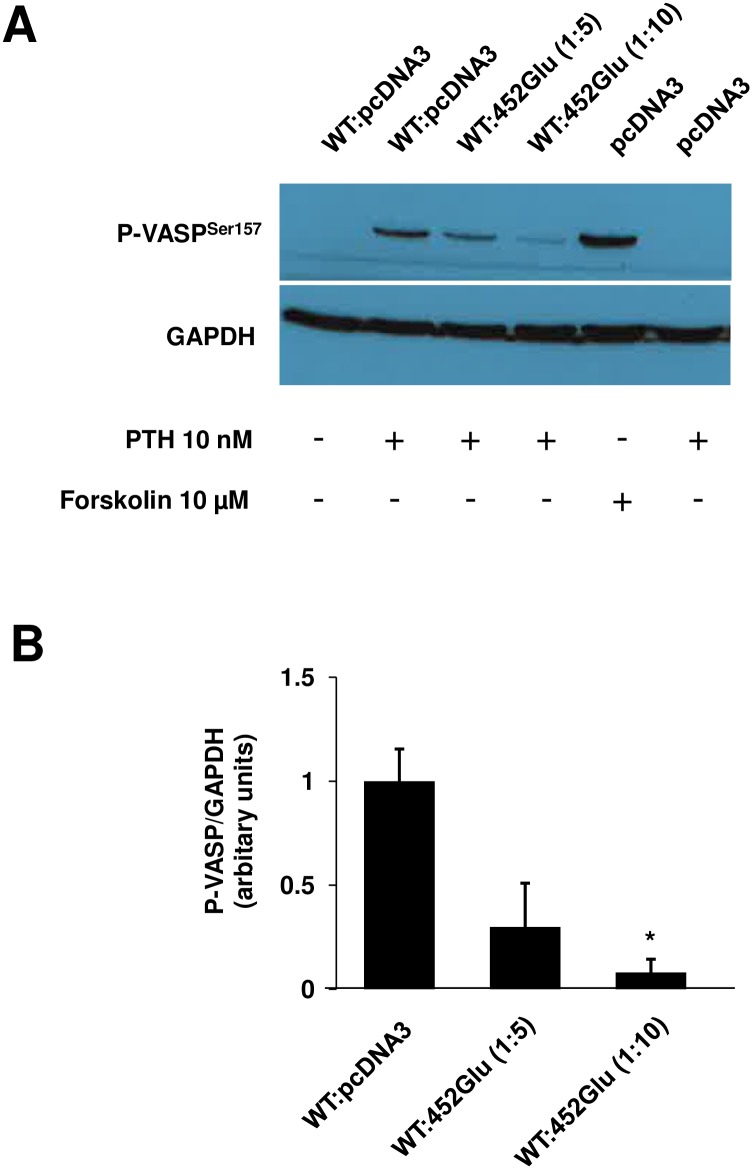
PTH induced VASP phosphorylation in HEK293 cells transfected with various ratio of PTH1R wild type and PTH1R/Gly453Glu mutant plasmids. (A) HEK293 cells were transfected with wild type and PTH1R/Gly452Glu plasmids in a ratio 1:5 (100 ng WT and 500 ng mutant) or 1:10 (100 ng WT and 1μg mutant) as indicated. As controls, pcDNA transfected cells and cells co-transfected with WT (100 ng) and pcDNA3 (1 μg) were used. 48 hours after transfection, cells were stimulated as indicated and lysed for Western immunoblots against P-VASP and GAPDH (loading control). Shown are representative blots of 3 independent experiments. (B) Bar graphs show quantification of phospho-VASP calculated with ImageJ software (n = 3). **p<0.05.

To identify whether mutant PTH1R/Gly452Glu has any dominant negative effect on wild type PTH1R activity, increasing amounts of PTH1R/Gly452Glu plasmid were cotransfected with a fixed amount of wild type PTH1R plasmid. After stimulation of cells with PTH, VASP phosphorylation was monitored. Western blot of VASP phosphorylation demonstrate that 5 or 10 times higher amounts of PTH1R/Gly452Glu plasmid transfected together with 100 ng wild type vector significantly reduced VASP phosphorylation in comparison to the effect of wild type PTH1R alone. This result documents a dominant negative effect of PTH1R/Gly452Glu mutant on the wild type receptor (Fig 3).

### Localization of PTH receptor variants

**Fig 4:**
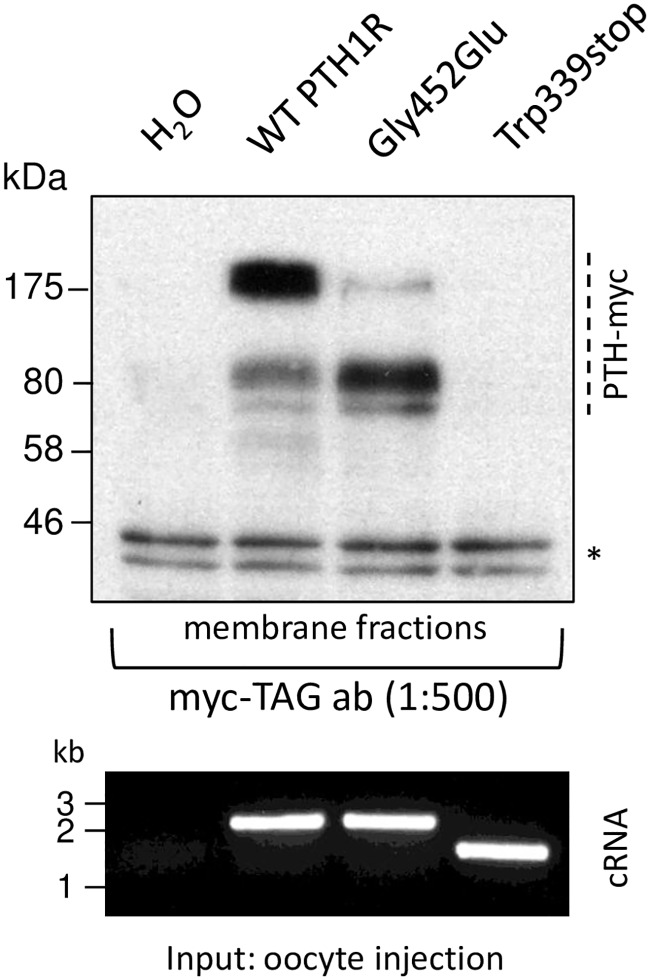
Membrane localization of PTH1R wild type and mutants. Membrane fractions of *Xenopus laevis* oocytes injected with cRNA of myc-tagged wild type (WT) and mutated PTH1R or H_2_O were analyzed by Western immunoblotting. As revealed by myc-tag antibody specific signals (as indicated on the right) were detected in preparations of PTH1R wild type and PTH1R/Gly452Gly whereas these protein bands were absent in samples of PTH1R/Trp339stop and H_2_O (negative control). Loading of equal amounts of protein is monitored by unspecific double bands (asterisk) in all samples. Quality and amount of cRNA injected into oocytes for heterologous expression of receptors is documented by RNA gel (lower panel).

To explore proper expression and targeting of wild type and mutated PTH1R we analysed myc-tagged proteins when expressed in *Xenopus* oocytes. Crude membrane fractions of oocytes, injected with identical amounts of cRNA of each receptor construct (Fig 4) were isolated and subjected to Western immunoblots. Specific signals, absent in H_2_O injected negative controls, were found in samples of PTH1R wild type, which indicates proper expression of the protein in oocytes. Also in preparations of mutant PTH1R/Gly452Glu specific protein bands of the receptor were detected. Prominent bands were detected at apparent molecular weight of >80 kDa and 175 kDa representing glycosylated monomeric and dimeric receptors. Although proteins in both samples display a different and inhomogeneous band pattern probably resulting from incomplete denaturation of sticky proteins, the specific signals document expression of PTH1R wild type and PTH1R/Gly452Glu mutant in membrane fractions in this expression system. However samples of PTH1R/Trp339stop do not show any signal with the antibody and thus document that the truncated protein is not located in membranes or not even expressed in the oocyte. As truncation at Trp339 disrupts the fourth transmembrane segment and eliminates the final three ones negative influence on the machinery of protein synthesis are very likely.

### Coupling of PTH receptor 1 to TRESK K_2_P channels

**Fig 5:**
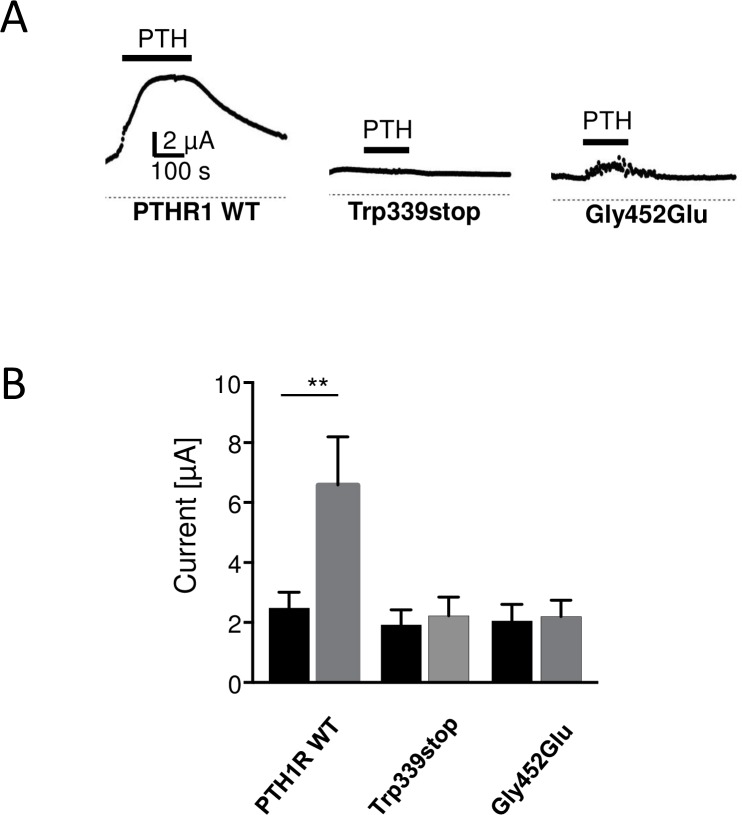
Coexpression of PTH1 receptor and TRESK in Xenopus oocytes. (A) Application of 100 nM PTH augments TRESK current amplitude 2.62-fold (left trace). Current augmentation was abolished upon co-expression of receptor mutants PTH1R/Trp339stop (middle trace) or PTH1R/Gly452Glu (right trace), respectively. (B) Bar graphs display TRESK current amplitudes before (black) and after PTH application (grey) for wild type PTH1R and the two PTH1R mutants.

In order to establish a physiological assay for PTH receptor activity we co-expressed the PTH1R with the tandem-pore potassium channel TRESK in *Xenopus* oocytes. TRESK currents are activated by G_αq_-coupled receptors via a phospholipase C-dependent pathway in a Ca^2+^-dependent manner. Activation of PTH1R with 100 nM PTH augmented TRESK currents by 2.62 ±0.43 fold (Fig 5, upper left; p<0.01; number of independent experiments (n), n = 17). In contrast, upon co-expression of the truncated receptor PTH1R/Trp339stop with TRESK channels, current amplitude was unchanged (1.14±0.05 fold; n = 13). The same was true for the second PTH1R mutant tested. Coupling of the mutant PTH1R/Gly452Glu to TRESK again failed to augment TRESK currents significantly (1.08±0.09 fold; n = 14). To elucidate whether the effect is specific for the exchange of Gly by Glu at position 452 we tested a conservative amino acid exchange. Remarkably substitution of glycine 452 in the wild type PTH1R to alanine did not significantly affect receptor function. In this case TRESK currents were significantly augmented by 2.23±0.45 fold (p<0.05, n = 18) and thus were comparable to the effect of wild type PTH receptors (data not shown). In summary, mutations leading to a truncated PTH receptor protein as well as a specific amino acid exchange at position 452, probably altering receptor structure, lead to loss of function of the PTH1R.

## Discussion

Primary failure of tooth eruption has been closely linked to heterozygous mutations in the gene encoding for PTH1 receptor [3]. We and others have identified until now more than 40 mutations causing deletions of the PTH1R, distinct nucleotide changes leading to exchange of single amino acids within the receptor or missense mutations causing changes of major parts of the amino acid sequence [6–8]. Although a wide range of algorithms and software tools are available which may predict the effects of amino acid changes in receptor molecules, until now functional effects of all these known PTH1R mutations were speculative and unclear.

In our investigations, we developed two new cell culture based functional assays that allow reliable testing of these various PTH1R mutants. In a first approach we tested two of the known PTH1R mutants isolated from patients with PFE [8]. Besides testing the mutant receptor on its own, our system also allows to mimic the heterozygous *in vivo* situation to detect functional interference between wild type and mutant PTH1R.

The correct eruption of teeth through the gingiva requires coordinated bone remodeling at the right time and place [17]. Mononuclear progenitor cells from the blood stream differentiate into bone forming osteoblastic cells under the influence of various signaling molecules including PTH, which plays a crucial regulatory role in this process [18]. Animal models using transgenic mice have elucidated the importance of intact PTH signaling for tooth eruption [17]. Missing links in PTH/PTH1R signaling pathways always resulted in failure of tooth eruption and major changes in bone development [19]. PTH1R mediates the effects of PTH in osteoblasts by stimulation of adenylate cyclase (AC) and by increase of cytosolic calcium concentration [20, 21]. Activation of AC increases intracellular cAMP in osteoblasts, subsequently activating cAMP-dependent protein kinases (PKAs) that phosphorylate and activate several cytoskeletal regulator proteins [21]. One major target of PKAs is the regulator protein VASP (vasodilator stimulated phosphoprotein) that coordinates both cell adhesion and cell migration in many cell types [22].

In our experimental model system we used HEK293 cells which supply all necessary signaling molecules of this PTH/cAMP/VASP signaling pathway except the PTH1R. Stimulation of HEK293 with PTH therefore did not result in any detectable increase in cAMP or VASP phosphorylation, whereas PTH-independent stimulation of AC via forskolin resulted in strong increase in cAMP and VASP phosphorylation. In this system, the transfection of human wild type PTH1R and stimulation of the cells with PTH showed good response with regard to intracellular cAMP signaling. This provides a new model system for testing the functional effects of the known PTH1R mutants derived from patients with PFE [3, 6, 7, 8].

PTH1R belongs to the group of seven-helical-transmembrane receptors. Binding of ligands to such receptors changes the configuration of the protein and mediates signaling effects inside the cell via binding of G-proteins to the intracellular domains of the receptors. Changes in the amino acid sequence of G-protein coupled receptors may have unexpected effects in functional testing [23]. Mutant receptors may not interfere with signaling of the wild type protein, however, dominant negative effects with complete suppression of receptor signaling as well as reduction of intracellular receptor signaling to a variable degree have been described in a variety of cases [23]. Even single amino acid mutations may result in loss as well as in gain of receptor function.

PTH1R is highly expressed on osteoblasts and renal tubular cells and regulates calcium homeostasis and bone formation. Besides PTH, PTH1R is further activated by PTHrP [10], a paracrine hormone that also regulates bone development and epithelial-mesenchymal interactions in developing teeth. A complete loss of PTH1R function results in lethal Blomstrand chondrodysplasia (BOCD, OMIM #215045), characterized by advanced endochondral bone maturation and premature ossification of all skeletal elements [2]. In EIKEN disease (MIM #600002), multiple epiphyseal dysplasia and retarded ossification are found [24]. Jansen chondrodysplasia (MIM #156400) originates from a mutation in PTH1R that results in permanent activation of the subsequent signaling pathways resulting in a dwarfism phenotype, micrognathia, disorganized metaphyseal areas of the bones, hypercalcemia and hypophosphatemia [25,26]. A further disease associated with PTH1R gene disorder is Ollier disease (OMIM #166000), an enchondromatosis with an asymmetric dwarfism and epiphyseal fusion abnormality [27].

Since a complete loss of PTH1R function is not compatible with live as seen in Bloomstrand osteochondropathia [28], this possibility of complete destruction of PTH signaling or strong dominant negative effect can be excluded for the mutants found in PFE patients. A single amino acid mutation in the intracellular tail of PTH1R has been described to interfere with cAMP signaling and cytosolic calcium transient [29]. Various autosomal dominant or recessive mutations in the PTH1R gene have been identified to be associated with clinical symptoms, however, functional data of these mutants are missing.

The clinical symptoms in PFE are discrete and limited to mainly posterior teeth and growth deficiency of the alveolar process in the affected region [3, 4]. Recently, an association with osteoarthritis was postulated [7]. In a first approach we tested the truncation mutant of PTH1R (PTH1R/Trp339stop) and a single amino acid exchange mutant (PTH1R/Gly452Glu), both were found in patients with PFE [8]. When transfected in HEK293 cells, the PTH1R/Gly452Glu mutant on its own was functionally inactive probably because the mutant protein was mostly retained within the cell and did not localize to the cell surface preventing the interaction with PTH ligand. On the other hand, our experiments show that PTH1R/Gly452Glu has a dominant negative effect on wild type PTH1R and thus the mutation of one allele not only reduces the amount of functional receptors but also negatively affects proper signaling of the PTH1R from the healthy allele. Consequently our data suggest that patients with heterozygous PTH1R mutations suffer from impaired receptor signaling due to gene dosage as well as dominant negative effects. This might explain the distinct clinical implications of heterozygous PTH1R mutations in patients with PFE. Besides the failure of tooth eruption in certain teeth and certain developmental time points, no further clinical signs of missing PTH signaling have been identified so far. Signaling of PTH regulates the two major intracellular responses, increase in cAMP and cytosolic calcium mainly independent of each other in the various cell systems of the organism. Therefore, the presence of PTH wild type receptor above a critical threshold level may be sufficient to allow normal cell development and function in most cell types despite the presence of a PTH1R mutant. However, in situations that may require maximal level of PTH signaling, the reduced PTH1R level in heterozygous PTH1R wild type/mutant cells may not achieve this critical functional degree of PTH signaling. This is consistent with our data suggesting that during tooth eruption, which is controlled by basal bone formation of dental osteoblasts, PTH-regulated signaling may drop below a critical threshold.

In a second approach we analyzed G_q_-coupled Ca^2+^ signaling of PTH receptors by electrophysiological recordings from Xenopus laevis oocytes. Prior, crude membrane fractions of cRNA-injected oocytes were subjected to Western immunoblots and demonstrated that RTH1R wild type and PTH1R/Gly452Glu mutant were properly expressed in the recombinant system. However, the truncation mutant PTH1R/Trp339stop was not detected. This failure of expression likely resulted from degradation of misfolded protein being selected by protein quality control [30].

For the electrophysiological assay K_2_P channel TRESK was chosen to analyze the function of G_q_-coupled seven-helix receptor signaling quantitatively [16]. Co-expression of the receptor together with TRESK in Xenopus oocytes allows to calculate the functional effect of different PTH1R mutations present in patients with PFE. In accordance with the results of biochemical experiments in HEK293 cells (Fig 2A) our electrophysiological recordings from *Xenopus* oocytes demonstrate that PTH1R/Trp339 and PTH1R/Gly452Glu are loss of function mutations. As evident from immunocytochemistry in HEK cells the point mutant was not targeted to the membrane whereas Western blots with oocytes show that the truncation mutant is not properly expressed.

In conclusion, our newly developed testing systems allow to obtain functional data on all described PTH1R mutants thereby providing a useful tool for the correct classification of the known genetic PTH1R variants found in patients with PFE. Further investigations are certainly necessary to reveal exact molecular mechanisms of interference of the PTH1R mutants with the wild type PTH1R signaling to elucidate the pathogenesis of PFE and to develop new therapeutic strategies for this disease.

## Supporting Information

S1 Table ANOVA statistics 1. (PZFX)

S2 Table ANOVA statistics 2. (PZFX)

S3 Table additional cAMP measurement data. (XLSX)
